# Inhibition of nuclear factor κB in the lungs protect bleomycin-induced lung fibrosis in mice

**DOI:** 10.1007/s11033-022-07185-8

**Published:** 2022-01-27

**Authors:** Devaang Thakur, Olivia Taliaferro, Madeleine Atkinson, Ryan Stoffel, Rakeshwar S. Guleria, Sudhiranjan Gupta

**Affiliations:** 1 Biomarkers and Genetics Core, VISN 17 Center of Excellence On Returning War Veterans, 4800 Memorial Drive, Waco, TX 76711 US; 2 Department of Biology, Baylor University, 101 Bagby Avenue, Waco, TX 76706 US; 3 Institute of Biomedical Studies, Baylor University, Waco, TX 76798 US; 4 Animal Facility, Baylor University, Baylor University, 101 Bagby Avenue, Waco, TX 76706 US

**Keywords:** NF-κB, Bleomycin, miRNA, Pulmonary, Fibrosis

## Abstract

**Background:**

Pulmonary fibrosis is a debilitating condition with limited therapeutic avenues. The pathogenicity of pulmonary fibrosis constitutes involvement of cellular proliferation, activation, and transformational changes of fibroblast to myofibroblasts. It is a progressive lung disease and is primarily characterized by aberrant accumulation of extracellular matrix proteins in the lungs with poor prognosis. The inflammatory response in the pathogenesis of lung fibrosis is suggested because of release of several cytokines; however, the underlying mechanism remains undefined. A genetic model is the appropriate way to delineate the underlying mechanism of pulmonary fibrosis.

**Methods and results:**

In this report, we have used cc-10 promoter based IκBα mutant mice (IKBM, an inhibitor of NF-κB) which were challenged with bleomycin (BLM). Compared to wild-type (WT) mice, the IKBM mice showed significant reduction in several fibrotic, vascular, and inflammatory genes. Moreover, we have identified a new set of dysregulated microRNAs (miRNAs) by miRNA array analysis in BLM-induced WT mice. Among these miRNAs, let-7a-5p and miR-503-5p were further analyzed. Our data showed that these two miRNAs were upregulated in WT-BLM and were reduced in IKBM-BLM mice. Bioinformatic analyses showed that let-7a-5p and miR-503-5p target for endothelin1 and bone morphogenic receptor 1A (BMPR1A), respectively, and were downregulated in WT-BLM mice indicating a link in pulmonary fibrosis.

**Conclusion:**

We concluded that inhibition of NF-κB and modulation of let-7a-5p and miR-503-5p contribute a pivotal role in pulmonary fibrosis and may be considered as possible therapeutic target for the clinical management of lung fibrosis.

## Introduction

Pulmonary fibrosis, a severe condition resulting from an injury to the lung parenchyma, causes increased proliferation and migration of fibroblasts and excessive accumulation of matrix proteins in the lung [1–3]. It is a progressive lung disease and primarily characterized by deposition of uncontrolled extracellular matrix (ECM) components including collagens, matrix metalloproteinases (MMPs) and tissue inhibitor of matrix proteinases (TIMPS) [4–6]. Fibroblasts play a vital role in fibrotic processes and are regulated by transforming growth factor-β (TGFβ) [7]. During the fibrotic process, the fibroblast becomes activated and transformed into myofibroblast by expressing α-smooth muscle actin (α-SMA or Acta) which is proliferative in nature. The key events of myofibroblast promotes apoptosis and fibrosis [8–10]. The myofibroblast is pivotal in pulmonary fibrosis and is linked to structural alteration caused by remodeling of ECM, eventually leading to tissue stiffness and, impaired lung function [11]. Treatment regimens for pulmonary fibrosis are limited. Therefore, identifying key molecule is required for the development of new targeted therapeutics for pulmonary fibrosis.

Accumulative evidence suggested that pro‐inflammatory and pro‐fibrotic cytokines are significantly involved in the pathogenesis of pulmonary fibrosis [12, 13]. Furthermore, it was suggested that excessive inflammation imparted the growth of fibroblasts in the formation of pulmonary fibrosis [14]. Nuclear Factor kappa B (NF-κB) is a pleiotropic transcription factor which regulates a set of genes responsible for both innate and adaptive immune responses [15, 16]. Apart from its’ vital role in immune modulation, NF-κB contributes a critical role in pathogenesis of many lung diseases including pulmonary fibrosis [17, 18]. We demonstrated previously that inhibition of NF-κB in the lungs using IκBα mutant (IKBM) gene, prevented monocrotaline-induced pulmonary hypertension in mice [19]. The IKBM mouse was made using Club (Clara) cell-10 (cc-10) promoter that drives IκBα mutant gene, an inhibitor of NF-κB. Although cc-10 promoter inhibits NF-κB primarily in airway epithelial cells, the existence of Clara cells was observed in the rodent lungs [20]. Therefore, we used this model to understand the role of NF-κB in lung (pulmonary) fibrosis which is currently unknown.

Recently, a small conserved non-coding RNA designated as microRNAs (miRNAs) were implicated in several vascular pathologies including pulmonary fibrosis [21–25]. Therapeutic strategies based on modulation of miRNA hold a great promise in disease pathology managements. MiRNAs are ~22 nucleotides in length which bind to the 3' untranslated region of specific target genes and suppress/inhibit their translation [26, 27]. Currently, NF-κB-mediated miRNA regulation in lung fibrosis remains unknown. In this conceptual setting, determination of miRNAs will provide new strategies to advance our current understanding of the pathogenesis of lung fibrosis. Although, a recent study has shown a signature of miRNA in bleomycin (BLM)-induced lung fibrosis [28]; a major gap remains in understanding the therapeutic benefits of miRNAs and NF-κB-linked miRNAs during pulmonary fibrosis. Despite significant progress in the understanding of pathological mechanisms of persistent fibrosis, effective therapeutic interventions are yet to be identified.

Here, we investigated whether NF-κB inhibition in the lungs using IKBM mice had any effect in BLM-induced pulmonary fibrosis. The generation of IKBM mouse was described previously [19, 29]. BLM is a cytotoxic agent used to treat cancer and induce inflammatory and fibrotic reactions in lungs mimicking idiopathic pulmonary fibrosis [30]. In this study, we hypothesized that the blocking of NF-κB in the lungs could mitigate BLM-induced lung fibrosis.

We determined the inflammatory and fibrotic gene expressions pattern in BLM-induced wild type (WT) and IKBM mice lungs. We report for the first time that BLM-induced pulmonary fibrosis is associated with NF-κB activation (RelA), upregulation of fibrotic genes, enhanced inflammatory response and altered expression of bone morphogenic protein receptor A (BMPR1A) in WT mice; and all these alterations were prevented in IKBM mice treated with BLM. Furthermore, several dysregulated miRNAs were identified in BLM-induced lung fibrosis and were restored in IKBM mice. Our data suggests that NF-κB-miR-503-5p-Col1A1-BMPR1A axis is an important regulator of pulmonary fibrosis and may serve as a target for promising therapeutic intervention.

## Materials and methods

### Generation of transgenic mice overexpressing the Iκ*B*α* mutant gene (IKBM)*

Generation of the cc-10 promoter driven IKBM mice has been previously described [29]. Age- and sex-matched wild-type (WT) mice of C57BL/6 background served as controls. Eight to ten-week-old mice (~25 g) were used for the experiments. The studies were conducted with the approval of Institutional Animal Care and Use Committee (IACUC) at Baylor University.

### Mouse model of bleomycin-induced pulmonary fibrosis

Eight to ten weeks old littermate WT mice (C57BL/6) and IKBM mice were used in this study. To induce pulmonary fibrosis, bleomycin (Sigma-Aldrich, St Louis, MO) was dissolved in sterile saline at 1 U/ml. After inducing anesthesia with intraperitoneal ketamine (80 mg/kg) and xylazine (15 mg/kg), bleomycin was injected intratracheally at a dose of 1.5 U/kg body weight. Control groups received the same volume of sterile saline only. Groups of 5–8 mice were sacrificed at day 21 after BLM treatment. The lungs were removed, washed with cold PBS and were snap frozen for mRNA and miRNA isolation. The tissue was used for miRNA microarray and real-time PCR analyses.

### Microarray processing and analysis

The miRNA isolation and preparation for MiRNA array were performed as described previously [31]. In brief, miRNA was isolated from the lungs of WT and IKBM mice using miRNeasy Kit (Qiagen, California, USA) as per manufacturer’s instructions. MiRNA array was performed and analyzed by LC Bioscience (Austin, TX, USA). The data was represented as a log_2_ value. Paired t-test or ANOVA were performed to find statistically significant differences between the groups and *P* < 0.05 was considered significant. The miRNA array data is MIAME compliant and was submitted to the gene expression omnibus (GEO) server at NCBI vide GEO no GSE155823.

### RNA extraction and quantitative real-time polymerase chain reaction (qRT-PCR)

RNA was extracted from the lungs of WT, IKBM, WT + BLM and IKBM + BLM mice using RNEasy kit (Qiagen, Valencia, CA, USA), following the manufacturer’s instructions. The qRT-PCR was performed as described previously [32, 33]. All primers used in the study were purchased from OriGene Technologies Inc. (Rockville, MD, USA) and follow manufacturer’s instruction for primer dilution and PCR amplification cycle.

### Statistical analysis

All experiments were performed using at least three to five separate mice lung tissue samples for each group. The RT-PCR analyses were performed in triplicates for each sample. All experiments were performed at least three times for each determination. Data was expressed as the means ± SE and was analyzed using one-way ANOVA using Prism 5.0 GraphPad software (GraphPad, San Diego, CA). *P* < 0.05 was considered as significant.

## Results

### BLM-induced lung fibrotic genes are prevented in IKBM mice

**Fig. 1:**
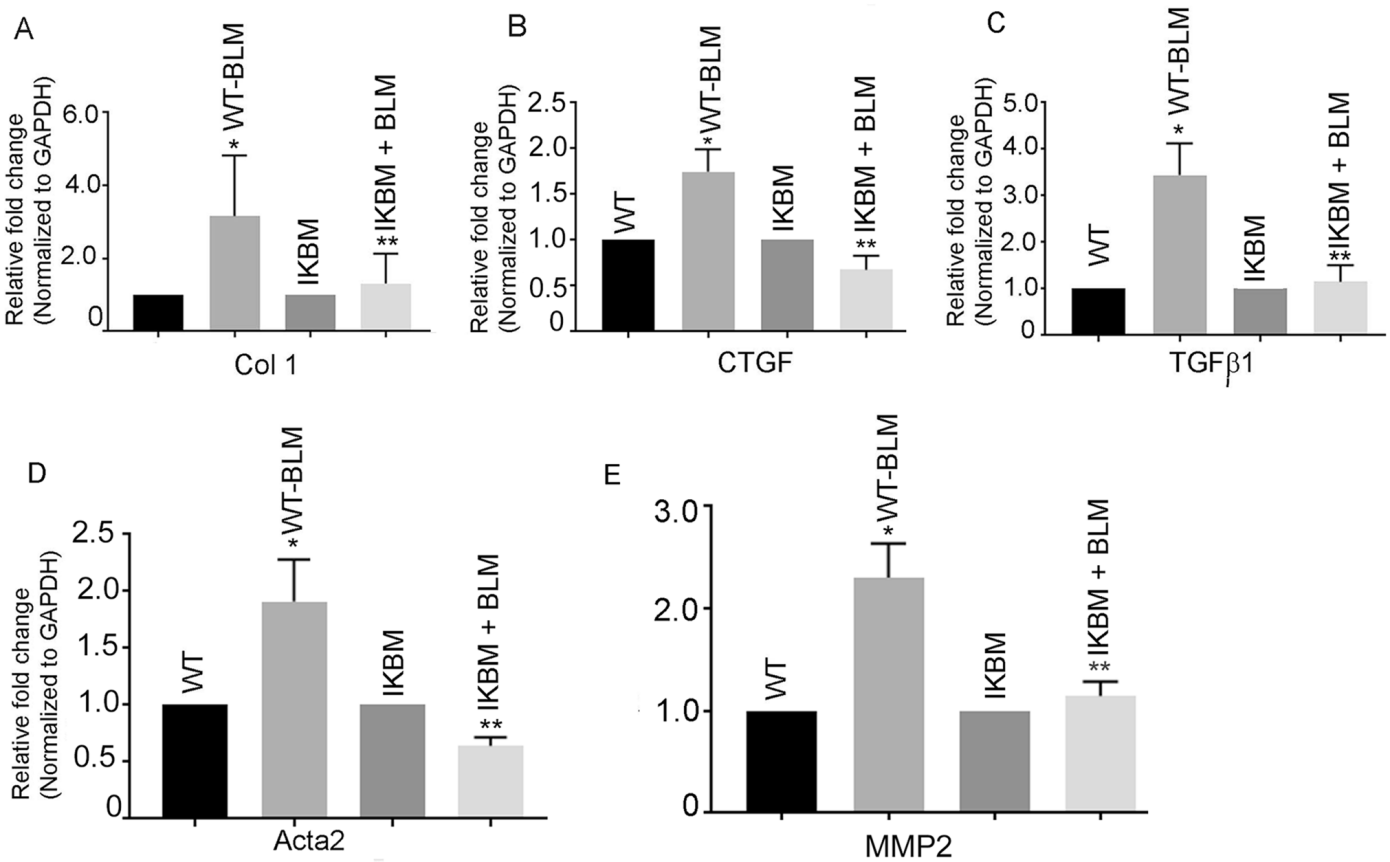
BLM-induced fibrotic genes are prevented in IKBM mice. The mRNA expression of fibrotic genes: Col A1a (**A**), CTGF (**B**), Tgfβ1(**C**), Acta2 (**D**) and MMP2 (**E**) in BLM-treated WT and IKBM mice was determined by quantitative RT-PCR. Data are expressed as means ± SE from 3 independent mice. *P < 0.05 compared with the WT mice. **P < 0.05 compared with the WT-BLM mice

The mRNA expression of several fibrotic genes, Col1A1, CTGF, MMP2, Acta2 and TGFβ1 was increased by 3.15 ± 0.83-, 1.74 ± 0.24-, 2.3 ± 0.19-, 1.91 ± 0.36 and 3.44 ± 0.39 (*P* < 0.05)-fold, respectively, in lungs of BLM-treated WT mice, compared with the untreated WT mice (Fig. 1A–E). The BLM-treated IKBM mice showed a significant down regulation of these genes, compared with BLM-treated WT mice (Col1A1, 1.31 ± 0.04, CTGF, 0.67 ± 0.15, MMP2, 1.14 ± 0.08-, Acta 2, 0.64 ± 0.07 and TGFβ1, 1.15 ± 0.2; *P* < 0.05) (Fig. 1A–E).

### BLM-induced inflammatory cytokines and RelA were prevented in IKBM mice

**Fig. 2:**
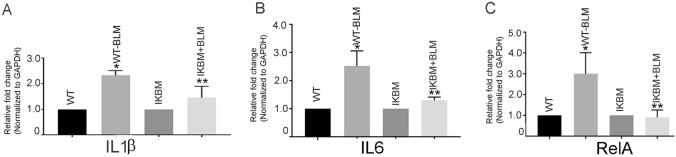
BLM-induced proinflammatory genes are prevented in IKBM mice. The mRNA expression of proinflammatory cytokine IL1β (**A**), IL6 (**B**), and RelA (**C**) in BLM-treated WT and IKBM mice was determined by quantitative RT-PCR. Data are expressed as means ± SE from 3 independent mice. *P < 0.05 compared with the WT mice. **P < 0.05 compared with the WT-BLM mice

The mRNA expression of proinflammatory cytokine genes; IL6, IL1β and RelA were increased by 2.52 ± 0.31-, 2.32 ± 0.06 and 3.01 ± 0.5 (*P* < 0.05)-fold, respectively, in the lungs of BLM-treated WT mice, compared with the untreated WT mice (Fig. 2A–C). The BLM-treated IKBM mice showed significant downregulation of the above genes, compared with the BLM-treated WT mice (1.30 ± 0.6-, 1.46 ± 0.25- and 0.91 ± 0.17-fold, *P* < 0.05, respectively (Fig. 2A–C).

### BLM treatment alters the expression of BMPR1A, Cav1, Edn1 and PECAM1 genes in the lungs

**Fig. 3:**
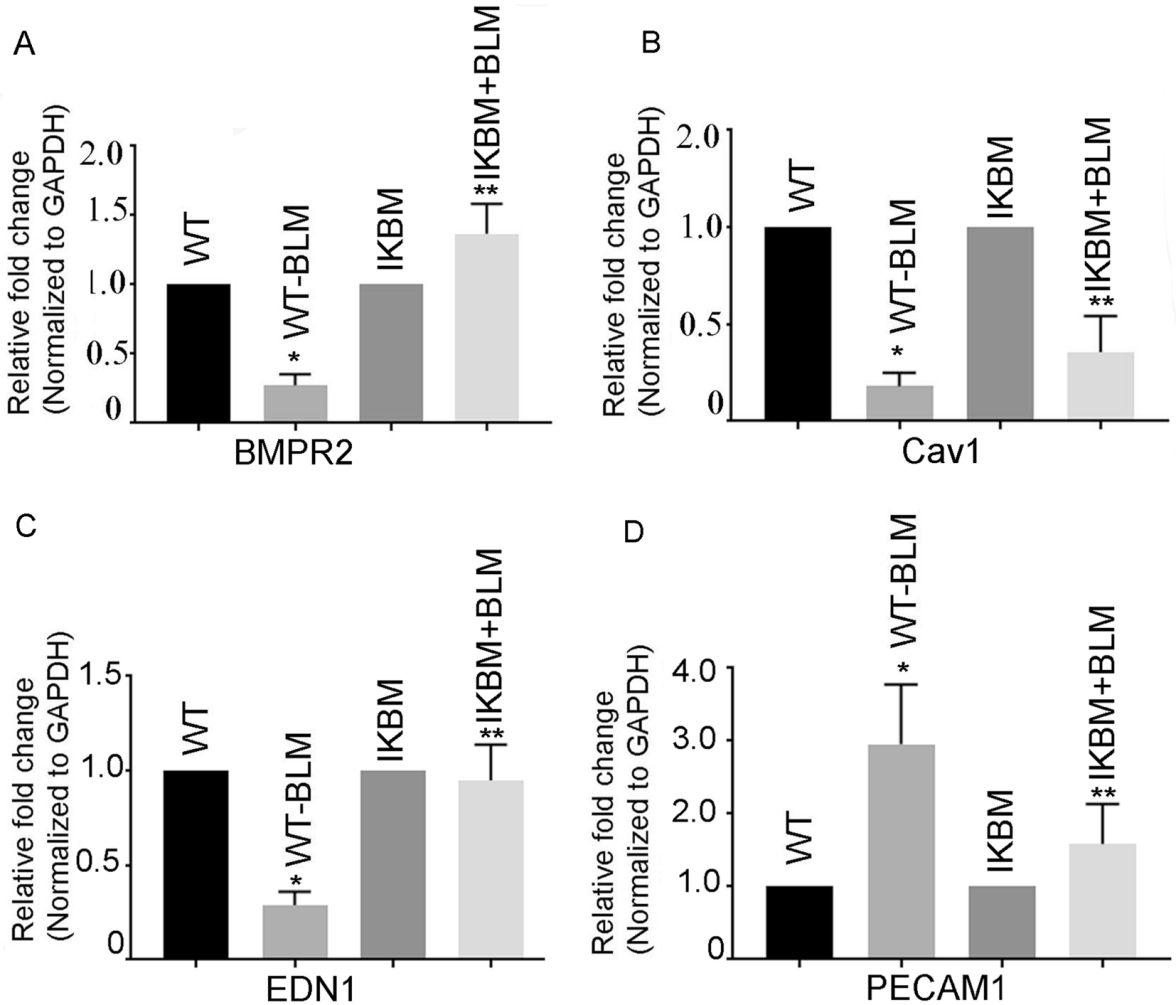
BLM-induced reduction of BMPR2, Cav1 and Edn1 are restored in IKBM mice. The mRNA expression of BMPR2 (**A**), Cav1 (**B**) and Edn1 (**C**) and PECAM1 (**D**) in BLM-treated WT and IKBM mice was determined by quantitative RT-PCR. Data are expressed as means ± SE from 3 independent mice. *P < 0.05 compared with the WT mice. **P < 0.05 compared with the WT-BLM mice

The mRNA expression of BMPR1A, Cav1 and Edn1 genes were reduced by 0.27 ± 0.04, 0.18 ± 0.804 and 0.95 ± 0.1-fold (*P* < 0.05), respectively, in BLM-treated WT mice, compared to untreated WT mice. The reduction of BMPR1A, Cav1 and Edn1 were restored in BLM-treated IKBM mice, compared with the BLM-treated WT mice (1.36 ± 0.12, 0.35 ± 0.01 and 0.29 ± 0.04, *P* < 0.05) (Fig. 3A–C). The restoration of these genes in IKBM group imply their regulation by NF-κB. Additionally, we have determined the expression of PECAM 1 gene. Our data showed an increase of 2.96 ± 0.40-fold in BLM-treated WT mice compared with the untreated WT mice (Fig. 3D). The IKBM mice showed a significant reduction of PECAM1 by 1.5 ± 0.27-fold (*P* < 0.05) compared to WT-BLM mice.

### Effect of expression of apoptotic gene in BLM-induced lung fibrosis

**Fig. 4:**
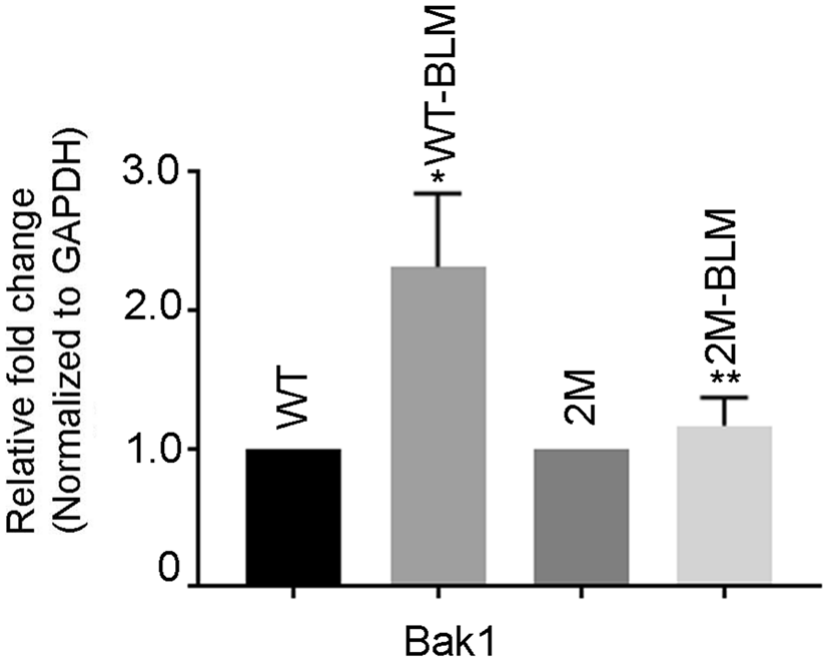
BLM treatment alters the expression of apoptotic family gene, Bak1 in the lung. The mRNA expression of pro-apoptotic gene Bak1 in BLM-exposed WT and IKBM mice were determined by quantitative RT-PCR. Data are expressed as means ± SE from 3 independent mice. *P < 0.05 compared with the WT mice. **P < 0.05 compared with the WT-BLM mice

Expression of the pro-apoptotic Bcl_2_ family gene, Bak is increased by 2.31 ± 0.30-fold in BLM-treated WT mice, compared with the untreated WT mice. The expression of Bak was reduced to 1.17 ± 0.11 (*P* < 0.05) in the BLM-treated IKBM mice (Fig. 4).

### MiRNA profiling in the lungs of BLM-treated WT and IKBM mice

**Fig. 5:**
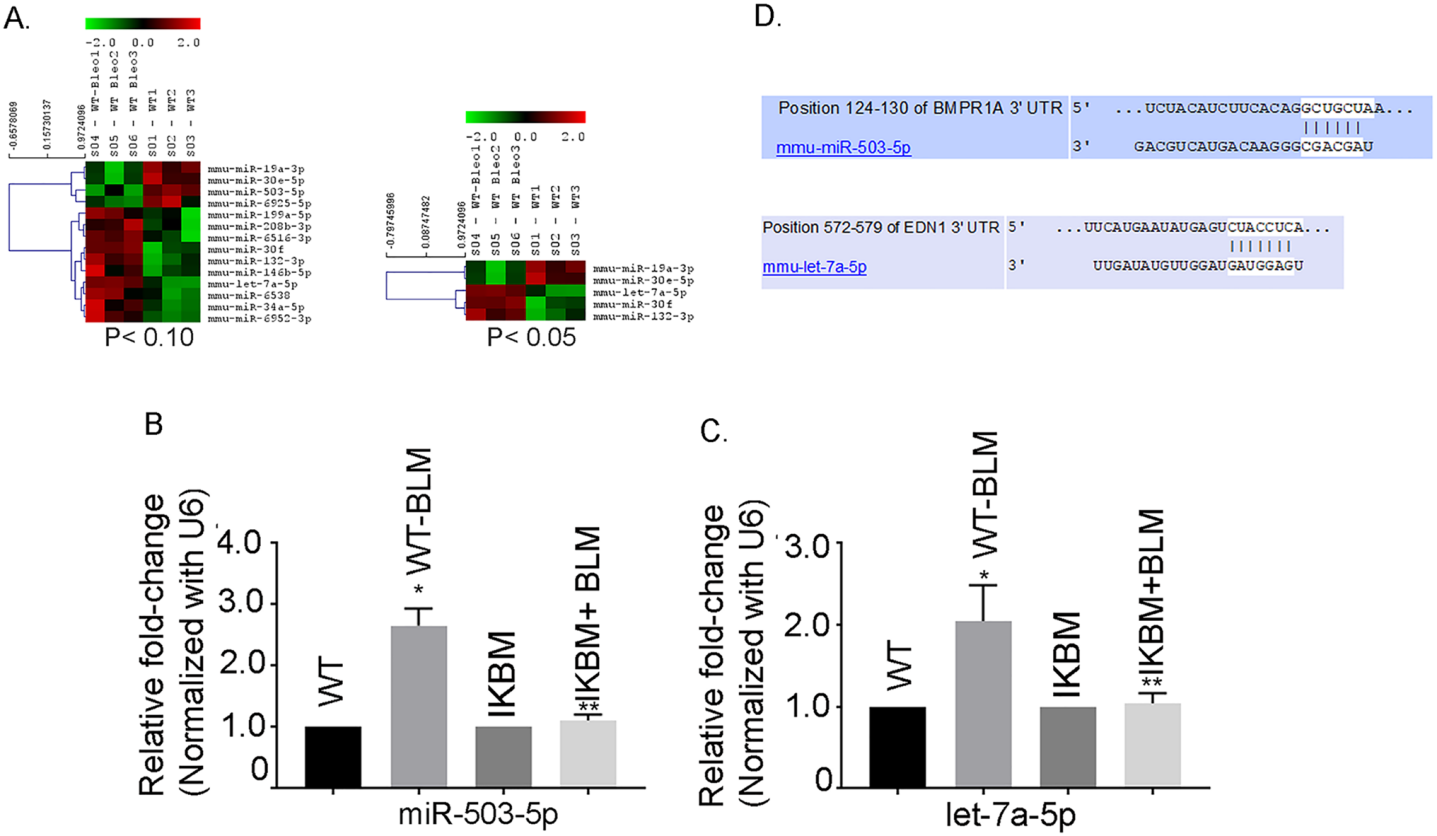
Heat map showing the expression of dysregulated miRNAs in WT and BLM treated mice lungs. **A** MiRNA microarray data was prepared using data from the miRNA microarray performed using samples from WT and BLM treated mice lungs and compared with untreated WT mice lungs. The signal difference (log2) is plotted versus the level of statistical significance (2log10 p-value). Color and spot diameter depicted information about mean expression level and percent detected, respectively, in static plots. **B** Validation of miRNAs in the lungs of WT and BLM treated mice. The mature miR-503-5p (**B**) and let-7a-5p (**C**) expressions were determined by qRT-PCR. U6 was used as an internal control. Data are expressed as means ± SE from 3 independent mice. *P < 0.05 compared with the WT mice. **P < 0.05 compared with the WT-BLM mice. **D** BMR1A and Edn1genes are the target for miR-503-5p and let-7a-5p. An alignment between BMPR1A and miR-503-5p as predicted by TargetScan 7.2 and miRBase analyses as shown in the upper panel by vertical bars. The sequence alignment of putative miR-503-5p and its targeting site on 3’-UTR of BMPR1A shows a high level of complementarity. An alignment between Edn1 and let-7a-5p as predicted by TargetScan 7.2 and miRBase analyses as shown in the bottom panel by vertical bars. The sequence alignment of putative let-7a-5p and its targeting site on 3’-UTR of Edn1 shows a high level of complementarity

MiRNA profiling analyses were performed using lungs of WT and IKBM mice treated with and without BLM by LC Sciences, Houston, TX. The arrays are based on Sanger miR Base release 14.0 databases. Color and spot diameter depicted information about mean expression level and percent detected were shown in the heat maps. For two-color experiments, the ratio of the two sets of detected signals (log_2_ transformed, balanced) and, the p values of the t-test were calculated (Fig. 5A). Our analysis revealed several novel dysregulated miRNAs in the lungs of WT-BLM group, compared to the untreated WT mice (Table 1).

**Table 1:** .

Rpter Index	Reporter Name	p-value	Group 1	Group 2	Log2 (G2/G1)
			WT-Bleo	WT	
			Mean	Mean	
3	mmu-let-7a-5p	2.94E-02	25,838	20,539	− 0.33
106	mmu-miR-30e-5p	4.34E-02	7604	8986	0.24
292	mmu-miR-199a-5p	8.98E-02	1091	863	− 0.34
223	mmu-miR-146b-5p	9.94E-02	811	481	− 0.75
183	mmu-miR-132-3p	2.50E-02	31	18	− 0.74
52	mmu-miR-19a-3p	3.11E-02	25	35	0.51
107	mmu-miR-30f	3.85E-02	64	26	− 1.29
1424	mmu-miR-6952-3p	5.90E-02	17	2	− 2.89
1301	mmu-miR-6538	6.16E-02	104	54	− 0.95
640	mmu-miR-503-5p	6.39E-02	61	98	0.68
1297	mmu-miR-6516-3p	8.36E-02	34	19	− 0.86
115	mmu-miR-34a-5p	9.31E-02	288	175	− 0.72
317	mmu-miR-208b-3p	9.48E-02	43	24	− 0.84
1371	mmu-miR-6925-5p	9.97E-02	19	41	1.12

### Validation of NF-κB-dependent miRNAs in BLM-induced lung fibrosis mouse model

The miR-503-5p and let-7a-5p expression were determined in the WT and IKBM mice lungs treated with BLM. The qRT-PCR analysis demonstrated a 2.65 ± 0.16-fold and 2.05 ± 0.25-fold increase in miR-503-5p and let-7a-5p, respectively (Fig. 5B, C); in the lungs of WT + BLM mice compared to untreated WT mice (*P* < 0.001). The expressions of both miRNAs were significantly reduced in BLM-treated IKBM mice (1.11 ± 0.05 and 1.04 ± 0.07) compared to WT + BLM treated mice (*P* < 0.05), indicating the regulation of miR-503-5p by NF-κB (Fig. 5 B, C).

To identify putative binding sites of miR‐503-5p in the 3′‐UTR, we used the miRNA target predicted search engine, TargetScan 7.2. As shown in miRNA: messenger RNA alignment analysis, 3′‐UTR of BMPR1A gene contained miR‐503-5p binding sites which were highly conserved among different species (Fig. 5D). The 7-mer sits on 128–134 nucleotide region of BMPR1A 3′UTR region. Our analysis of upregulation of miR-503-5p as shown in Fig. 5A and downregulation of BMPR1A as shown in Fig. 3A suggested a potential link between these molecules. Our data also demonstrated an upregulation of let-7a-5p shown in Fig. 5C and down regulation of Edn1 shown in Fig. 3C suggested a potential connection between them.

## Discussion

Our results demonstrate for the first time that inhibition of NF-κB in the lungs attenuated BLM-induced lung fibrosis. The mechanism is likely to be associated with restoration of BMPR1A-Acta2-Col I-axis molecules and, inhibition of the inflammatory response. Additionally, miRNA array analysis revealed the involvement of the novel miRNAs, miR-503-5p and let-7a-5p in BLM-induced lung fibrosis. There are few reports indicating that inhibition of NF-κB is effective in pulmonary fibrosis. However, there is no report showing a direct link between genetic blockade of NF-κB in the lungs and pulmonary fibrosis [34–36]. This report provides for the first time that the genetic inhibition of NF-κB in the lungs prevents the BLM-induced lung fibrosis.

Our study evaluated the hallmark genes for fibrosis like Col1A1, Acta2, CTGF, MMP2 and TGFβ1, which are critical in extracellular matrix (ECM) deposition, a key process in fibrosis. All the above genes are significantly reduced in IKBM mice challenged with BLM indicating that they are regulated by NF-κB. It is reported that CTGF has NF-κB binding element in the promoter region, and this may suggest a direct regulation of CTGF by NF-κB [37]. The regulation of Col1A1 by NF-κB may involve the interaction with other transcription factors binding sites that were present in Col1A1 promoter since Col1A1 promoter did not show any NF-κB binding element [38]. Also, our study showed a significant increase in TGFβ1 mRNA expression. It is known that TGFβ has many physiological functions which are associated with airway remodeling and fibrosis and is a triggering factor for ECM deposition [39]. The TGFβ pathway is pivotal in the pathogenesis of lung fibrosis [40]. It has been reported that deletion of the TGFβ receptor type II is protective against BLM-induced pulmonary fibrosis in mice [41]. Moreover, TGFβ activation triggers fibroblast activation leading to the formation of myofibroblast by irreversible acquisition of alpha-smooth muscle actin, Acta2 protein, another important player in fibrosis [42]. Differentiation of lung fibroblasts to myofibroblasts is a key event in the pathogenesis of several fibrotic diseases including pulmonary fibrosis [43]. Our study shows that inhibition of NF-κB in the lungs significantly reduced Acta2 expression in BLM treated IKBM mice indicating an effective therapeutic approach for the treatment of pulmonary fibrosis.

NF-κB is a key regulator of inflammatory response. The transcription of many inflammatory cytokines, such as TNFα, IL6, IL1β and TGFβ, are vital in development of lung fibrosis [44, 45]. Our data showed that both IL1β and RelA was significantly reduced in BLM-induced IKBM mice indicating a NF-κB-mediated regulation. Activation of inflammatory molecules particularly TNFα and TGFβ promotes inflammation; damaging ECM protein components and triggering myofibroblast differentiation that leads to the development of lung fibrosis [46, 47].

Bone morphogenic proteins are the member of TGFβ family members and contributed a critical role in pulmonary hypertension, a proliferative vascular disease [19, 48, 49]. But the role of BMPR1A in BLM-induced pulmonary fibrosis is currently unknown. Previously, we have shown that monocrotaline-induced pulmonary hypertension was attenuated in IKBM mice by restoring BMPR2 (in part) indicated NF-κB- mediated regulation [19]. The current study also showed a significant reduction in BMPR2 in BLM-induced pulmonary fibrosis and was restored in IKBM mice indicating a NF-κB-mediated regulation. There is a report for inappropriate NF-κB signaling in BMPRII-deficient pulmonary arterial smooth muscle cells and increased IL6 secretion [50]. Our data also revealed a significant increase in IL6 expression that was reduced in IKBM mice which may correlate an association between BMPR1A-IL6 and NF-κB in pulmonary fibrosis.

Furthermore, the study revealed an upregulation of Acta2 in BLM treated WT mice which was reduced in IKBM mice. The phenomenon of endothelial-to-mesenchymal transition (EndoMT) is critical in lung fibrosis [51–53] which is characterized by acquiring mesenchymal phenotype, such as α-SMA (Acta2). This is the first report showing NF-κB regulated this critical phenotypic switch in the pathogenesis of pulmonary fibrosis. The study also showed Cav1, a highly expressed membrane protein in the lungs, is downregulated in BLM-induced pulmonary fibrosis. The Cav1 contributes an essential role in tissue repair and fibrosis by modulating ECM turnover [54]. Furthermore, Cav1 is shown to be a critical regulator of idiopathic lung fibrosis [55]. Our data is consistent with a previous observation that reduced Cav1 promotes fibrosis [55]. This new finding shows that restoration of Cav1 in IKBM mice suggest a potential connection between NF-κB and Cav1 in lung fibrosis which is unknown but needs further investigation.

Finally, we have identified a panel of dysregulated miRNAs in BLM-treated WT mice and the current study validated let-7a-5p and miR-503-5p as potential miRNAs in pulmonary fibrosis. Interestingly, these two miRNAs were upregulated in BLM treated WT group and were reduced in BLM treated IKBM group suggested a possible regulation by NF-κB. Modulation of miRNA is suggested to be a possible therapeutic target in several fibrotic diseases including pulmonary fibrosis [21–25, 56]. Previously, we have demonstrated NF-κB-mediated miR-130a regulation in TGFβ1 stimulated lung microvascular remodeling [33]. However, the association between let-7a-5p, miR-503-5p and NF-κB in pulmonary fibrosis is unknown. Our study provides evidence for the first time that let-7a-5p and miR-503-5p may be regulated by NF-κB. Furthermore, bioinformatic tool for miRNA target analysis showed that miR-503-5p has potential target for BMPR1A. Downregulation of BMPR1A and upregulation of miR-503-5p in BLM treated WT mice may suggest a possible underlying mechanism of pulmonary fibrosis. The identification of miR-503-5p and let-7a-5p along with their target genes are preliminary observations documented in the current form of the study. Further research is underway to delineate detail mechanism in pulmonary fibrosis.

In conclusion, our study identifies the importance of NF-κB-miR-503-5p-Col1A1-BMPR1A- axis and provide new mechanistic information in BLM-induced lung fibrosis. The cytokine released followed by EndMT provide new insights for the progression of lung fibrosis. Our data further suggests that let-7a-5p and miR-503-5p play a critical role in lung fibrosis and may be considered as possible therapeutic targets.

## Data Availability

Data will be made available on reasonable request.
